# A More Intense Examination of the Intensity of Physical Activity in People Living with Chronic Obstructive Pulmonary Disease: Insights from Threshold-Free Markers of Activity Intensity

**DOI:** 10.3390/ijerph191912355

**Published:** 2022-09-28

**Authors:** Andrew P. Kingsnorth, Alex V. Rowlands, Benjamin D. Maylor, Lauren B. Sherar, Michael C. Steiner, Mike D. Morgan, Sally J. Singh, Dale W. Esliger, Mark W. Orme

**Affiliations:** 1 Diabetes Research Centre, University of Leicester, Leicester General Hospital, Leicester LE5 4PW, UK; 2 NIHR Leicester Biomedical Research Centre, Leicester LE5 4PW, UK; 3 School of Sport, Exercise and Health Sciences, Loughborough University, Loughborough LE11 3TU, UK; 4 Department of Respiratory Sciences, University of Leicester, Leicester LE1 9HN, UK; 5 Centre for Exercise and Rehabilitation Science, NIHR Leicester Biomedical Research Centre-Respiratory, Leicester LE3 9QP, UK

**Keywords:** COPD, exercise capacity, physical activity

## Abstract

Physical activity (PA) intensity of people living with chronic obstructive pulmonary disease (COPD) is typically evaluated using intensity thresholds developed in younger, healthier populations with greater exercise capacity and free from respiratory symptoms. This study therefore compared (i) PA differences between COPD and non-COPD controls using both traditional intensity thresholds and threshold-free metrics that represent the volume and intensity of the whole PA profile, and (ii) explored the influence of exercise capacity on observed differences. Moderate-to-vigorous physical activity (MVPA), average acceleration (proxy for volume, mg) and intensity distribution of activity were calculated for 76 individuals with COPD and 154 non-COPD controls from wrist-worn ActiGraph accelerometry. PA profiles representing the minimum intensity (acceleration, mg) during the most active accumulated 5–960 min were plotted. Estimated VO_2peak_ and relative intensity were derived from the incremental shuttle walk test distance. Compared to the non-COPD control group, individuals with COPD recorded fewer MVPA minutes (59 vs. 83 min/day), lower overall waking activity (29.1 vs. 36.4 mg) and a poorer waking intensity distribution (−2.73 vs. −2.57). Individuals with COPD also recorded a lower absolute intensity (acceleration, mg) for their most active 5–960 min, but higher intensity relative to their estimated exercise capacity derived from the ISWT. People with COPD have a lower volume and absolute intensity of PA than controls but perform PA at a higher relative intensity. There is a need to move away from absolute intensity thresholds, and towards personalised or relative-intensity thresholds, to reflect reduced exercise capacity in COPD populations.

## 1. Introduction

For people living with chronic obstructive pulmonary disease (COPD), breathlessness at rest, exercise intolerance and reduced levels of physical activity (PA) are commonplace [1,2,3], and are associated with greater risk of hospitalisation and premature mortality [4]. Improving exercise capacity has always been a core therapeutic target (e.g., of pulmonary rehabilitation) but, more recently, the importance of increasing the PA of people living with COPD has been recognised [5]. However, studies including measures of PA suggest that improving exercise capacity does not necessarily result in an increase in PA [6,7,8].

Data from accelerometer-based measures of PA intensity are typically analysed using thresholds to determine time spent at a given intensity. Although these thresholds are derived from calibration studies (e.g., Freedson et al. [9] and Troiano et al. [10]) in younger and healthier adults, they are commonly used to examine the intensity of PA of people with COPD [11]. Current evaluation of PA in COPD using a ‘one size fits all’ approach is in direct contradiction to the principle of individually prescribed exercise training during pulmonary rehabilitation.

Translating the individually prescribed nature of pulmonary rehabilitation exercise training (predicated on relative intensity) into the evaluation of free-living PA requires adjustment for exercise capacity. To date, classification of PA intensity has been limited to intensity categories which can mask meaningful differences across the intensity distribution. This is particularly true in chronic disease groups such as COPD, typically characterised by reduced exercise capacity, meaning any given relative PA intensity will occur at a lower absolute intensity in those with COPD compared with healthy adults, e.g., moderate intensity PA in people with COPD will likely be classified as light intensity PA when accelerometer thresholds from healthy populations are used. Thus, the limitations of current approaches means that purposeful PA at an intensity that is of moderate intensity for a person living with COPD may be missed completely when analysed using intensity thresholds generated from healthy populations. This may also mask potentially important changes in PA following interventions. Continuous markers of PA intensity that do not rely on thresholds (i.e., threshold-free metrics) such as the intensity gradient and average acceleration capture the whole intensity distribution, are comparable across devices and populations [12,13], and may contribute to a more clinically appropriate evaluation of PA in COPD.

Accordingly, the aims of this study were to compare (i) PA differences between COPD and non-COPD controls using both traditional intensity thresholds and threshold-free metrics that represent the volume and intensity of the whole PA profile, and (ii) to explore the influence of exercise capacity on any observed differences.

## 2. Materials and Methods

### 2.1. Dataset

Data were obtained as part of the Physical Activity and Respiratory Health (PhARaoH) study [14]. Individuals with a General Practitioner confirmed diagnosis of COPD aged between 40–75 years were recruited via the Primary Care Research Network and non-COPD controls were recruited from the community across Leicestershire and Rutland. All participants provided informed consent, with ethical approval granted from National Health Service Research Ethics Committee East Midlands Nottingham-2 (13-EM-0389).

### 2.2. Measurements

#### 2.2.1. Descriptive Measures

A full description of the measures taken within this study is outlined within Orme et al. [14]. In brief, height was measured using a portable stadiometer, and weight and percent body fat via bioelectrical impedance (Tanita MC780MA; Tanita Corporation, Tokyo, Japan), with height and weight used to derive body mass index (kg/m^2^). Waist circumference was obtained via the average of three measurements from around the mid-point between the lowest rib and iliac crest [15]. Forced spirometry (MicroLab MK8 spirometer; CareFusion, San Diego, CA, USA) was used to assess lung function in accordance with the American Thoracic Society/European Respiratory Society guidelines [16].

#### 2.2.2. Physical Activity

Participants were asked to wear an ActiGraph wGT3X-BT (ActiGraph, LLC, Pensacola, FL, USA) for seven consecutive days on their non-dominant wrist. Participants were instructed to wear the device at all times except water-based activities. Devices were initialised to collect data at 100 Hz and .gt3x accelerometry files were downloaded via ActiLife 6.10.1 (ActiGraph, Pensacola, FL, USA).

All accelerometry files were converted into .csv before being processed via GGIR version 2.4-2 [17]. The average magnitude of dynamic acceleration corrected for gravity (Euclidean Norm minus one g) was calculated in milli-gravitational units (mg) and averaged over 5 s epochs. Accelerometry files were auto-calibrated and excluded if post calibration errors were >0.01 g or if participants recorded fewer than 3 days of valid wear, defined as ≥16 h/day [18]. All accelerometer outcomes were expressed as the mean of all valid days. To enable calculation of time spent inactive, sleep duration was calculated using the automated sleep detection (HDCZA sleep detection algorithm [19]).

Average magnitude of dynamic wrist acceleration (Euclidean norm minus one) wrist worn ActiGraph thresholds were used to determine waking time spent inactive (<44.8 mg), in light (44.8–100.5 mg), moderate (100.6–428.7 mg), and vigorous PA (≥428.8 mg) [20,21]. Time accumulated in bouts of MVPA lasting 1- < 5, 5- < 10 and ≥10 min was also calculated, requiring ≥80% of each bout to be above the set threshold (GGIR Part 5) [13]. The configuration file information is included in Appendix A, Table A1.

#### 2.2.3. Threshold-Free Metrics: Average Acceleration and Intensity Gradient

The average acceleration represents overall PA across the waking day, measured in miligravitational units (mg) and is a proxy for volume of activity. The intensity gradient describes the association between the log of PA intensity and the log of time spent at that intensity during the waking day (GGIR Part 5). As the intensity of activity increases the time spent at that intensity decreases, thus the value is always negative. Therefore, a more negative (steeper) intensity gradient indicates proportionately more time is spent inactive and at lower PA intensities, while a less negative (flatter) intensity gradient indicates proportionately more time spent at higher PA intensities [22].

Visual illustrations of PA profiles were generated using MX radar plots in R (version 4.1.0 [R Foundation for Statistical Computing, Vienna, Austria]) using freely available code available from GitHub [23], and were interpreted as described in Rowlands et al. [13]. MX radar plots describe the intensity (acceleration, mg) above which the most active minutes of the day are accumulated with M corresponding with the time duration studied and X being the intensity; e.g., an M5 of 100 mg means that, over the entire day, 5 min were accumulated above an intensity of 100 mg [13]. The higher the value, the more intense the PA. MX values were generated in part 2 of GGIR for the most active 5 min to 960 min (16 h).

Plots were generated to compare profiles between controls and people living with COPD, Differences between groups can be hard to see where MX values are low (e.g., the intensity of periods 8 h [480 min] or longer [M480]); thus, standardised MX metrics were also generated and illustrated on a radar plot, calculated relative to the mean and standard error of the mean of the whole sample.

#### 2.2.4. Exercise Capacity

Exercise capacity was estimated using the incremental shuttle walk test (ISWT [24]), which was developed specifically for individuals with COPD, and correlates with other, more traditional laboratory assessment of oxygen uptake [25]. The test estimated peak oxygen uptake (VO_2peak_) was calculated using the best distance attained from two tests and participant body mass (kg) [26].

#### 2.2.5. Exercise Capacity and Physical Activity

A relative-intensity (relative to the maximum achieved in a sustained progressive exercise test) MX radar plot was also plotted for both the control and COPD groups. This indicates the percent of predicted maximum acceleration for the most active accumulated 5 min to 960 min per day, e.g., M5 = most active 5 min per day. Predicted maximum acceleration during the ISWT for each individual was derived from the speed (km/h) of the last fully achieved stage during the incremental shuttle walk test entered into the regression equation to predict acceleration from walking speed, age, height, mass and sex outlined by Dawkins et al. (reference equation obtained from author) [27]. Relative intensity of the most active accumulated 5–960 min per day was then calculated as MX values expressed as a percentage of an individual’s predicted maximum acceleration during the ISWT.

### 2.3. Statistical Analyses

Linear regression models were calculated using R (version 4.1.0 [R Foundation for Statistical Computing, Vienna, Austria]) to compare continuous characteristics of the sample between groups. Models were adjusted for age and sex with significance set at *p* < 0.05. Exercise capacity (estimated VO_2peak_) was also included within further models to ascertain if any differences in PA persist after adjustment for exercise capacity.

## 3. Results

### 3.1. Participant Characteristics

Individuals with COPD were statistically significantly older, had a greater waist circumference, lower estimated VO_2peak_ and worse lung function (Table 1). From the 259 participant accelerometer files available to be processed, 230 files were taken forward into the analyses. Files were removed for having <3 valid days (n = 3), a calibration error above threshold (n = 16) or not having a valid ISWT result (n = 10).

Summary and statistical differences are displayed within Table 2 and Table 3, respectively. Accelerometer wear compliance was comparable and high in both groups with 99.97% of participants recording ≥ 5 valid days (COPD = 6.0 ± 0.1 vs. controls = 5.9 ± 0.4, *p* > 0.05) and on average 983 min (16.4 h) of waking wear duration per day (COPD = 1000.0 ± 108.8 vs. controls = 976.6 ± 102.9, *p* > 0.05).

### 3.2. Differences in Physical Activity between COPD and Controls

Individuals with COPD spent more waking time inactive (777 ± 109 vs. 712 ± 103 min/day, *p* = 0.006), with less moderate (59 ± 34 vs. 81 ± 39 min/day, *p* = 0.007) and vigorous (1 ± 1 vs. 2.0 ± 3 min/day, *p* = 0.008) PA compared to controls. Controls also spent significantly more time in bouts of MVPA lasting ≥ 5 min/day (*p* < 0.012), but not lasting 1–5 min/day (10 ± 10 vs. 14 ± 10 min/day, *p* > 0.05).

The overall waking profile of PA was poorer in people with COPD compared to non-COPD controls, with a lower volume (average acceleration, 29.1 ± 8.7 vs. 36.4 ± 9.1 mg, *p* < 0.001) and poorer intensity distribution (a more negative (steeper) intensity gradient (−2.73 ± 0.21 vs. −2.57 ± 0.19, *p* < 0.001).

**Figure 1:**
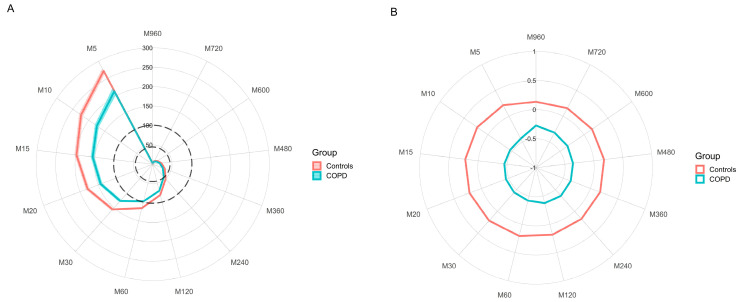
Radar plots representing the MX metrics for both the control and COPD groups. The metrics indicate the minimum level of acceleration (mg) for the most active durations outlined from 5 min to 960 min, e.g., M5 = most active 5 min per day. Coloured thick lines indicate the average value per group and the shaded portions either side of the lines indicates the standard error of the mean (Plot (**A**) only). The black dotted lines (Plot (**A**)) represent the threshold in mg for inactivity (44.8 mg [21] and moderate to vigorous physical activity (100.6 mg threshold [20]. Standardised differences in MX values (Plot (**B**)) were calculated relative to the mean and standard deviation (SD) of the whole sample.

The MX metrics calculated for both groups are displayed within Figure 1 for acceleration values (Plot A) and standardised differences relative to the mean and SD of the sample (Plot B). Individuals with COPD recorded a lower acceleration value for their most active minutes for all durations displayed (5–960 min). Differences between the two groups approximated 0.5 SD across the PA profile, irrespective of the intensity.

### 3.3. Physical Activity and Exercise Capacity

**Figure 2:**
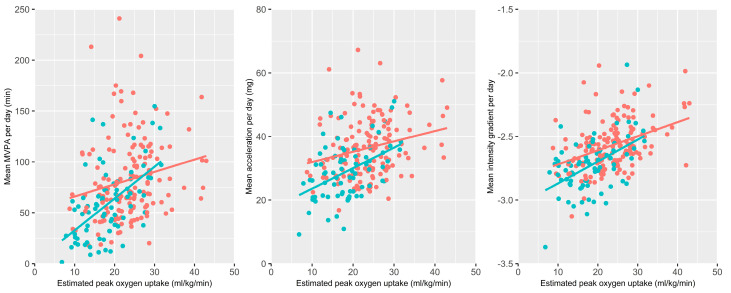
Scatter plots displaying the relationship between estimated peak oxygen uptake (VO_2peak_) against moderate to vigorous physical activity (MVPA), average acceleration and intensity gradient. Colouring indicates COPD diagnoses (blue = COPD & red = control) and lines represents linear regression lines.

Figure 2 displays mean MVPA, average intensity and intensity gradient against estimated VO_2peak_. Regression lines show a tendency for PA fitness slopes to be steeper in individuals with COPD, suggesting that the differences in PA between groups were mainly evident in those of lower fitness.

**Figure 3:**
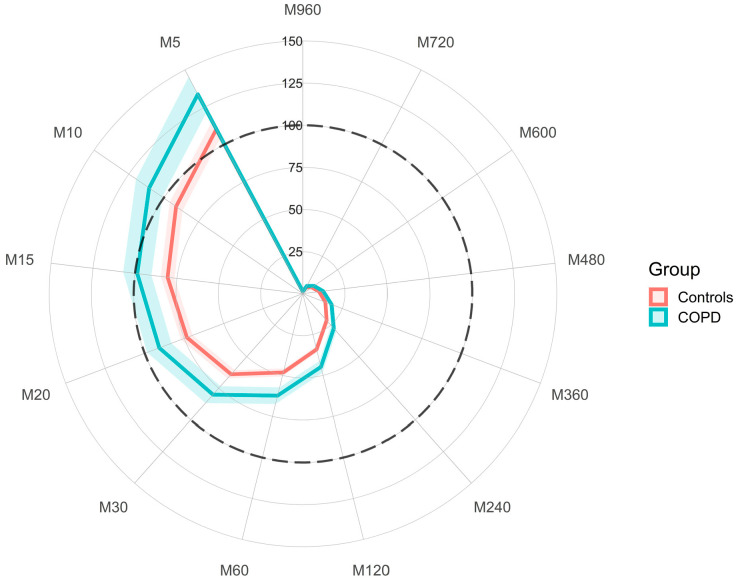
Radar plot representing the relative-intensity-adjusted MX metrics for both the control and COPD groups. The metrics indicate the intensity of the most active 5 min to 960 min of the day (e.g., M5 = most active 5 min per day) expressed as the percent of predicted maximum acceleration attained during the ISWT (black dotted line). The predicted maximum acceleration was derived by using the speed (km/h) from the last fully achieved stage during the incremental shuttle walk test and then using the equation as outlined by Dawkins et al. [27]. Coloured thick lines indicate the average value per group and the shaded portions either side of the lines indicates the standard error of the mean.

When the intensity of the most active periods of the day were plotted relative to predicted peak acceleration achieved in the ISWT (Figure 3), despite the COPD group having on average lower acceleration values per duration (Figure 1), accelerations were at a higher relative intensity than the control group. This suggests that, relative to their capacity, the COPD group complete their activity at a higher intensity relative to their exercise capacity than the controls for the majority of the day.

After statistical adjustment for exercise capacity, differences between groups for PA variables derived from absolute thresholds turned non-significant, apart from inactive time (*p* = 0.042) and MVPA bouts in 10+ minutes (*p* = 0.004). However, in contrast, differences between the continuous threshold-free PA markers were attenuated, but remained significantly different between groups (Model 2, Table 3).

## 4. Discussion

### 4.1. Summary of Main Findings

This study confirms that the overall waking profile of PA is poorer in individuals with COPD compared to controls, and that this difference persists after controlling for exercise capacity. However, examining the intensity of the most active minutes in relation to personalised predicted maximum acceleration during the ISWT revealed that individuals with COPD conduct a lower volume of physical activity but at a higher relative intensity than controls. These findings support the notion that current commonly used absolute intensity thresholds are unsuitable for COPD populations. There is a need to move away from absolute current intensity thresholds, and towards personalised or relative-intensity thresholds, to reflect the reduced exercise capacity evident in some populations. This goes beyond COPD, including populations that are older or not as healthy as those commonly used to develop accelerometer thresholds.

To the best of our knowledge, this is the first use of the average acceleration and intensity gradient metrics within a COPD cohort, and the findings are comparable with studies of other populations. An earlier study comparing physical activity data over the 24 h day between office workers and those with cardiometabolic disease reported greater group differences between the intensity gradient (0.17 (present study), 0.19 [28]) and average acceleration (7.7 mg (present study), 5.2 mg [28]), to those reported in the present study. The group differences in average acceleration translate to approximately 3850 steps/day [29], and 2600 steps/day in the present study and earlier study, respectively. The greater group difference in the present study may be reflective of the overall mild-moderate airway obstruction of our sample, compared with previous studies [30,31].

Exercise capacity and PA are both key targets during pulmonary rehabilitation [32]. It was notable that when the intensity of physical activity was expressed relative to the personalised intensity attained during the maximum speed in the ISWT, the COPD group appeared to be active at higher relative intensities than the control group. Indeed, relative intensity of the most active accumulated 5 (control) and 15 (COPD) minutes across the day was greater than the maximum acceleration predicted in the ISWT that required a sustained continuous effort over 10–15 min.

The elevated relative intensity suggests that patients with COPD are performing short activities at or above the maximum achieved within a progressive exercise test. Where absolute intensity (acceleration) is low (e.g., Figure 1A), this suggests that the capacity of these COPD patients is reduced to an extent that activities necessary for normal daily living are taxing the patients to near maximal levels. Alternatively, if high relative intensity is accompanied with moderate-to-high absolute intensity this may suggest high physical functioning on a daily basis. More work is needed to understand how these behaviours translate into physiological changes over time, or whether this relationship indicates that patients require additional behavioural credit when summarising behaviour into volume of activity. Threshold-free metrics could provide further insight by facilitating the capture of change in both relative and absolute intensity of PA following exercise interventions. Additionally, the generation of disease specific normative PA values may also be helpful to unearth the absolute and relative differences in PA intensity profiles between groups.

As exercise intolerance and physical inactivity are commonplace in COPD populations, as well as linked to increased risk of premature mortality [4], it is important to examine PA in the context of the interplay between these clinical characteristics. The observed differences in exercise-capacity-adjusted MX metrics and the attenuation of relationships using PA derived from thresholds after adjusting for exercise capacity again raises questions about the suitability of commonly used absolute thresholds for patients with COPD. The use of threshold-free metrics such as intensity gradient and average acceleration could therefore be utilised within this population group, considering their increased discrimination to determine PA differences, after adjusting for exercise capacity into account.

As individuals with COPD had a lower exercise capacity than controls, differences in PA intensity between these groups may not only be driven by differences in behaviour, but also representative of what intensity means for those with reduced exercise capacity. Just as individuals with COPD apply different meanings to sedentary behaviour [33], PA also takes on an intrinsic meaning seldom reflected in the quantification of this behaviour. Activity derived from thresholds can and still should be used due to their widespread understanding of the outputs such as minutes of MVPA, but in keeping with individually prescribed exercise during pulmonary rehabilitation, it may be appropriate to explore relative-intensity thresholds to explore PA in the context of their exercise capacity limits [34]. Indeed, despite recent advancements in accelerometer data processing, absolute PA intensity thresholds remain derived from younger and healthier adults with greater exercise capacity than COPD populations.

### 4.2. Strengths and Limitations

The main strength of this study is the assessment of PA using threshold-free metrics, using gravitational units comparable across devices and populations, and comparison to non-COPD controls. These results further support the use of threshold-free intensity metrics when assessing PA within chronic disease populations, as they do not require arbitrary thresholds which have not been validated within the same population. In this study, the majority of the sample had mild-to-moderate airflow obstruction. As there were observable differences in PA with the controls, results are likely to be more pronounced for people with more severe airflow obstruction and respiratory symptoms. We included all suitable data within the analyses, but acknowledge there was a greater number of control participants and of a different sex split than compared to the COPD group.

Additionally, the measure of exercise capacity was derived by the ISWT, which is a widely used population specific measure, but requires distance units to be converted into an estimation of peak VO_2_ via a validated equation (rather than being directly calculated) for individuals with COPD. Deriving individualised or relative PA intensity thresholds directly from maximal exercise tests (e.g., ISWT) should be explored in future studies.

## 5. Conclusions

To the authors’ knowledge, this is the first study to apply threshold-free intensity metrics on wrist-worn ActiGraph data within a COPD population. The overall PA profile was poorer for individuals with COPD compared with non-COPD controls; with a lower average intensity, a poorer intensity gradient reflecting lower intensity during the most active minutes of the day, greater inactive minutes and fewer MVPA minutes per day. Individuals with COPD and reduced exercise capacity spent less time in PA matching the required threshold to be considered as MVPA, demonstrating the limitation of using absolute thresholds in this population. Statistical adjustment for exercise capacity attenuated but did not fully negate the observed differences in PA between groups differing by exercise capacity, whereas adjustment of the intensity of free-living PA for personalised predicted peak acceleration during the ISWT suggested that individuals with COPD performed PA at a higher relative intensity than non-COPD controls. There is a need to move away from current absolute intensity thresholds. Direct adjustments, such as individually calibrating PA intensity using acceleration measured during maximal walking tests, are needed to provide further insight into the physical activity of people with COPD.

## Figures and Tables

**Table 1:** Characteristics of the sample, reported as mean (SD) unless otherwise stated.

	COPD (N = 76)		Controls (N = 154)		*p*
**Demographics**					
Age (years)	65.9	(6.8)	56.6	(8.9)	***
Sex (female/male)	28/48%	37/63%	91/63	59/41%	
**Body composition**					
Body mass index (kg/m^2^)	28.2	(5.1)	26.8	(5.1)	
Percentage body fat (%) ^a^	28.8	(9.0)	29.6	(7.6)	
Waist circumference (cm)	99.4	(14.0)	90.2	(13.8)	*
**Exercise capacity**					
ISWT distance (m)	395.7	(160.3)	523.8	(174.2)	***
Estimated VO_2peak_ (mL/kg/min)	18.4	(6.2)	23.6	(6.6)	***
**Respiratory health** ^b^					
FEV1 (L)	2.0	(0.8)	2.5	(0.7)	***
FEV1 % predicted	72.2	(21.5)	94.0	(19.9)	***
FVC (L)	3.6	(1.0)	3.4	(0.9)	
FEV1/FVC	55.1	(12.8)	73.3	(7.4)	***

Notes: Values for certain variables were not available for all participants; ^a^ Controls n = 151 ^b^ COPD n = 75, con n = 153; abbreviations (FEV_1_ = forced expiratory volume, FVC = forced vital capacity, ISWT = incremental shuttle walk test); significant differences between groups *p* < 0.001 = *** and *p* < 0.05 = * (adjusted for age and sex, apart from age).

**Table 2:** Accelerometry summary data, reported as mean (SD) unless otherwise stated.

	COPD		Controls	
Inactive (min/day)	777.4	(108.8)	711.9	(102.9)
Light activity (min/day)	163.4	(52.6)	182.2	(50.7)
Moderate activity (min/day)	58.7	(34.2)	80.6	(39.4)
Vigorous activity (min/day)	0.6	(1.1)	1.8	(3.1)
MVPA (min/day)	59.3	(34.9)	82.5	(40.0)
MVPA bouts 1–5 (min/day)	10.2	(9.9)	13.5	(10.4)
MVPA bouts 5–10 (min/day)	3.9	(4.9)	6.1	(6.5)
MVPA bouts 10+ (min/day)	6.6	(12.1)	13.4	(17.4)
Average acceleration (mg)	29.1	(8.7)	36.4	(9.1)
Intensity gradient	−2.73	(0.21)	−2.57	(0.19)

Notes: abbreviations (MVPA = moderate to vigorous physical activity, Standard deviation = SD); intensity gradient is specified to 2 dp; bouts are defined as <5, 5- < 10 and ≥10 min; waking average acceleration and intensity gradient are represented in the table.

**Table 3:** Multiple linear regression models representing the differences between groups for physical activity variables.

	**Model 1**			
	**B**	**SE**	**R^2^**	***p***
Inactive (min/day)	−45.22	16.28	0.12	**
Light activity (min/day)	12.17	8.01	0.07	
Moderate activity (min/day)	16.10	5.95	0.09	**
Vigorous activity (min/day)	1.10	0.41	0.06	**
MVPA (min/day)	17.20	6.03	0.10	**
MVPA bouts 1–5 (min/day)	1.80	1.61	0.05	
MVPA bouts 5–10 (min/day)	2.40	0.95	0.03	*
MVPA bouts 10+ (min/day)	10.16	2.48	0.08	***
Average acceleration (mg)	5.73	1.42	0.14	***
Intensity gradient	0.12	0.03	0.15	***
	**Model 2**			
	**B**	**SE**	**R^2^**	***p***
Inactive (min/day)	−34.32	16.77	0.14	*
Light activity (min/day)	9.45	8.32	0.26	
Moderate activity (min/day)	9.84	6.01	0.14	
Vigorous activity (min/day)	0.46	0.40	0.18	
MVPA (min/day)	10.30	6.06	0.16	
MVPA bouts 1–5 (min/day)	0.26	1.63	0.09	
MVPA bouts 5–10 (min/day)	1.64	0.97	0.06	
MVPA bouts 10+ (min/day)	7.33	2.49	0.14	**
Average acceleration (mg)	3.85	1.40	0.22	**
Intensity gradient	0.07	0.03	0.27	*

Notes: each line represents the results of the COPD grouping variable as an independent predictor for each behavioural variable; abbreviations (MVPA = moderate to vigorous physical activity); model 1 has been adjusted by age and sex whilst model 2 has been further adjusted for exercise capacity (estimated VO_2peak_ (mL/kg/min)); R^2^ is represented as adjusted; bouts are defined as <5, 5- < 10 and ≥10 min; significant differences between groups *p* < 0.001 = ***, *p* < 0.01 = ** and *p* < 0.05 = *; COPD group coded = 1; waking average acceleration and intensity gradient are represented in the table.

## Data Availability

Not applicable.

## Appendix A

**Table A1:** Config file.

Argument	Value	Context
R_version	R version 4.1.0 (2021-05-18)	not applicable
backup.cal.coef	retrieve	Calibration, Feature extraction, Epoch size, Time zone
chunksize	1	Calibration, Feature extraction, Epoch size, Time zone
configtz	c()	Calibration, Feature extraction, Epoch size, Time zone
dayborder	0	Calibration, Feature extraction, Epoch size, Time zone
do.anglex	FALSE	Calibration, Feature extraction, Epoch size, Time zone
do.angley	FALSE	Calibration, Feature extraction, Epoch size, Time zone
do.anglez	TRUE	Calibration, Feature extraction, Epoch size, Time zone
do.bfen	FALSE	Calibration, Feature extraction, Epoch size, Time zone
do.cal	TRUE	Calibration, Feature extraction, Epoch size, Time zone
do.dev_roll_med_acc_x	FALSE	Calibration, Feature extraction, Epoch size, Time zone
do.dev_roll_med_acc_y	FALSE	Calibration, Feature extraction, Epoch size, Time zone
do.dev_roll_med_acc_z	FALSE	Calibration, Feature extraction, Epoch size, Time zone
do.en	FALSE	Calibration, Feature extraction, Epoch size, Time zone
do.enmo	TRUE	Calibration, Feature extraction, Epoch size, Time zone
do.enmoa	FALSE	Calibration, Feature extraction, Epoch size, Time zone
do.hfen	FALSE	Calibration, Feature extraction, Epoch size, Time zone
do.hfenplus	FALSE	Calibration, Feature extraction, Epoch size, Time zone
do.lfen	FALSE	Calibration, Feature extraction, Epoch size, Time zone
do.lfenmo	FALSE	Calibration, Feature extraction, Epoch size, Time zone
do.mad	FALSE	Calibration, Feature extraction, Epoch size, Time zone
do.roll_med_acc_x	FALSE	Calibration, Feature extraction, Epoch size, Time zone
do.roll_med_acc_y	FALSE	Calibration, Feature extraction, Epoch size, Time zone
do.roll_med_acc_z	FALSE	Calibration, Feature extraction, Epoch size, Time zone
dynrange	c()	Calibration, Feature extraction, Epoch size, Time zone
hb	15	Calibration, Feature extraction, Epoch size, Time zone
lb	0.5	Calibration, Feature extraction, Epoch size, Time zone
minloadcrit	72	Calibration, Feature extraction, Epoch size, Time zone
myfun	c()	Calibration, Feature extraction, Epoch size, Time zone
n	4	Calibration, Feature extraction, Epoch size, Time zone
print.filename	FALSE	Calibration, Feature extraction, Epoch size, Time zone
printsummary	FALSE	Calibration, Feature extraction, Epoch size, Time zone
windowsizes	c(5, 900, 3600)	Calibration, Feature extraction, Epoch size, Time zone
acc.metric	ENMO	General parameters
bout.metric	4	General parameters
config_file_in_outputdir	C:/Users/………	General parameters
data_cleaning_file	c()	General parameters
datadir	D:/………..	General parameters
desiredtz		General parameters
do.bfx	FALSE	General parameters
do.bfy	FALSE	General parameters
do.bfz	FALSE	General parameters
do.hfx	FALSE	General parameters
do.hfy	FALSE	General parameters
do.hfz	FALSE	General parameters
do.lfx	FALSE	General parameters
do.lfy	FALSE	General parameters
do.lfz	FALSE	General parameters
do.parallel	TRUE	General parameters
do.report	c(2,4,5)	General parameters
do.sibreport	FALSE	General parameters
do.zcx	FALSE	General parameters
do.zcy	FALSE	General parameters
do.zcz	FALSE	General parameters
excludefirst.part4	FALSE	General parameters
excludelast.part4	FALSE	General parameters
f0	1	General parameters
f1	260	General parameters
frag.metrics	c()	General parameters
GGIRversion	2.4-2	General parameters
HASIB.algo	vanHees2015	General parameters
HASPT.algo	HDCZA	General parameters
HASPT.ignore.invalid	FALSE	General parameters
i	30	General parameters
idloc	1	General parameters
includedaycrit.part5	0.666667	General parameters
interpolationType	1	General parameters
longitudinal_axis	c()	General parameters
LUX_cal_constant	c()	General parameters
LUX_cal_exponent	c()	General parameters
LUX_day_segments	c()	General parameters
LUXthresholds	c(0, 100, 500, 1000, 3000, 5000, 10,000)	General parameters
maxNcores	c()	General parameters
minimum_MM_length.part5	23	General parameters
minimumFileSizeMB	2	General parameters
mode	c(1, 2, 3, 4, 5)	General parameters
MX.ig.min.dur	10	General parameters
outputdir	C:/Users/…….	General parameters
overwrite	FALSE	General parameters
part5_agg2_60seconds	FALSE	General parameters
qwindow_dateformat	%d-%m-%Y	General parameters
relyonguider	FALSE	General parameters
rmc.bitrate	c()	General parameters
rmc.check4timegaps	FALSE	General parameters
rmc.col.acc	c(1, 2, 3)	General parameters
rmc.col.temp	c()	General parameters
rmc.col.time	c()	General parameters
rmc.col.wear	c()	General parameters
rmc.dec	.	General parameters
rmc.desiredtz		General parameters
rmc.doresample	FALSE	General parameters
rmc.dynamic_range	c()	General parameters
rmc.firstrow.acc	c()	General parameters
rmc.firstrow.header	c()	General parameters
rmc.format.time	%Y-%m-%d %H:%M:%OS	General parameters
rmc.header.length	c()	General parameters
rmc.header.structure	c()	General parameters
rmc.headername.recordingid	c()	General parameters
rmc.headername.sf	c()	General parameters
rmc.headername.sn	c()	General parameters
rmc.noise	FALSE	General parameters
rmc.origin	########	General parameters
rmc.sf	c()	General parameters
rmc.unit.acc	g	General parameters
rmc.unit.temp	C	General parameters
rmc.unit.time	POSIX	General parameters
rmc.unsignedbit	TRUE	General parameters
Sadeh_axis		General parameters
save_ms5raw_format	csv	General parameters
save_ms5raw_without_invalid	TRUE	General parameters
selectdaysfile	c()	General parameters
sensor.location	wrist	General parameters
sleeplogsep	,	General parameters
sleepwindowType	SPT	General parameters
storefolderstructure	FALSE	General parameters
studyname	c()	General parameters
week_weekend_aggregate.part5	FALSE	General parameters
anglethreshold	5	Parameters sleep detection
constrain2range	TRUE	Parameters sleep detection
ignorenonwear	TRUE	Parameters sleep detection
timethreshold	5	Parameters sleep detection
colid	1	Parameters sleep period time detection with or wihout sleeplog
coln1	1	Parameters sleep period time detection with or wihout sleeplog
criterror	4	Parameters sleep period time detection with or wihout sleeplog
def.noc.sleep	1	Parameters sleep period time detection with or wihout sleeplog
do.visual	TRUE	Parameters sleep period time detection with or wihout sleeplog
excludefirstlast	FALSE	Parameters sleep period time detection with or wihout sleeplog
includenightcrit	16	Parameters sleep period time detection with or wihout sleeplog
loglocation	c()	Parameters sleep period time detection with or wihout sleeplog
nnights	c()	Parameters sleep period time detection with or wihout sleeplog
outliers.only	TRUE	Parameters sleep period time detection with or wihout sleeplog
relyonsleeplog	c()	Parameters sleep period time detection with or wihout sleeplog
sleeplogidnum	TRUE	Parameters sleep period time detection with or wihout sleeplog
boutcriter.in	0.9	Parameters time-use variables
boutcriter.lig	0.8	Parameters time-use variables
boutcriter.mvpa	0.8	Parameters time-use variables
boutdur.in	c(1, 10, 30)	Parameters time-use variables
boutdur.lig	c(1, 5, 10)	Parameters time-use variables
boutdur.mvpa	c(1, 5, 10)	Parameters time-use variables
excludefirstlast.part5	FALSE	Parameters time-use variables
save_ms5rawlevels	FALSE	Parameters time-use variables
threshold.lig	44.8	Parameters time-use variables
threshold.mod	100.6	Parameters time-use variables
threshold.vig	428.8	Parameters time-use variables
timewindow	WW	Parameters time-use variables
boutcriter	0.8	Study design, Parameters descriptive analysis
closedbout	FALSE	Study design, Parameters descriptive analysis
do.imp	TRUE	Study design, Parameters descriptive analysis
do.part3.pdf	TRUE	Study design, Parameters descriptive analysis
epochvalues2csv	FALSE	Study design, Parameters descriptive analysis
hrs.del.end	0	Study design, Parameters descriptive analysis
hrs.del.start	0	Study design, Parameters descriptive analysis
iglevels	c(0, 25, 50, 75, 100, 125, 150, 175, 200, 225, 250, 275, 300, 325, 350, 375, 400, 425, 450, 475, 500, 525, 550, 575, 600, 625, 650, 675, 700, 725, 750, 775, 800, 825, 850, 875, 900, 925, 950, 975, 1000, 1025, 1050, 1075, 1100, 1125, 1150, 1175, 1200, 1225, 1250, 1275, 1300, 1325, 1350, 1375, 1400, 1425, 1450, 1475, 1500, 1525, 1550, 1575, 1600, 1625, 1650, 1675, 1700, 1725, 1750, 1775, 1800, 1825, 1850, 1875, 1900, 1925, 1950, 1975, 2000, 2025, 2050, 2075, 2100, 2125, 2150, 2175, 2200, 2225, 2250, 2275, 2300, 2325, 2350, 2375, 2400, 2425, 2450, 2475, 2500, 2525, 2550, 2575, 2600, 2625, 2650, 2675, 2700, 2725, 2750, 2775, 2800, 2825, 2850, 2875, 2900, 2925, 2950, 2975, 3000, 3025, 3050, 3075, 3100, 3125, 3150, 3175, 3200, 3225, 3250, 3275, 3300, 3325, 3350, 3375, 3400, 3425, 3450, 3475, 3500, 3525, 3550, 3575, 3600, 3625, 3650, 3675, 3700, 3725, 3750, 3775, 3800, 3825, 3850, 3875, 3900, 3925, 3950, 3975, 4000, 8000)	Study design, Parameters descriptive analysis
ilevels	c()	Study design, Parameters descriptive analysis
includedaycrit	16	Study design, Parameters descriptive analysis
IVIS.activity.metric	2	Study design, Parameters descriptive analysis
IVIS_epochsize_seconds	NA	Study design, Parameters descriptive analysis
IVIS_windowsize_minutes	60	Study design, Parameters descriptive analysis
M5L5res	10	Study design, Parameters descriptive analysis
maxdur	9	Study design, Parameters descriptive analysis
mvpadur	c(1, 5, 10)	Study design, Parameters descriptive analysis
mvpathreshold	100.6	Study design, Parameters descriptive analysis
ndayswindow	7	Study design, Parameters descriptive analysis
qlevels	c(0.333333333333333, 0.5, 0.583333333333333, 0.666666666666667, 0.75, 0.833333333333333, 0.916666666666667, 0.958333333333333, 0.979166666666667, 0.986111111111111, 0.989583333333333, 0.993055555555556, 0.996527777777778, 0.998611111111111, 0.999305555555556)	Study design, Parameters descriptive analysis
qM5L5	c()	Study design, Parameters descriptive analysis
qwindow	c(0, 24)	Study design, Parameters descriptive analysis
strategy	1	Study design, Parameters descriptive analysis
TimeSegments2ZeroFile	c()	Study design, Parameters descriptive analysis
window.summary.size	10	Study design, Parameters descriptive analysis
winhr	5	Study design, Parameters descriptive analysis
dofirstpage	TRUE	Visual report
viewingwindow	1	Visual report
visualreport	FALSE	Visual report
